# An Optical Fiber-Based Sensor Array for the Monitoring of Zinc and Copper Ions in Aqueous Environments

**DOI:** 10.3390/s140203077

**Published:** 2014-02-17

**Authors:** Steven Kopitzke, Peter Geissinger

**Affiliations:** Chemistry and Biochemistry Department, University of Wisconsin-Milwaukee, 3210 N. Cramer Ave. Milwaukee, WI 53201, USA; E-Mail: kopitzke@uwm.edu

**Keywords:** optical fiber, fluorescent sensor, zinc, copper, optical time of flight detection, sensor array

## Abstract

Copper and zinc are elements commonly used in industrial applications as aqueous solutions. Before the solutions can be discharged into civil or native waterways, waste treatment processes must be undertaken to ensure compliance with government guidelines restricting the concentration of ions discharged in solution. While currently there are methods of analysis available to monitor these solutions, each method has disadvantages, be it high costs, inaccuracy, and/or being time-consuming. In this work, a new optical fiber-based platform capable of providing fast and accurate results when performing solution analysis for these metals is described. Fluorescent compounds that exhibit a high sensitivity and selectivity for either zinc or copper have been employed for fabricating the sensors. These sensors demonstrated sub-part-per-million detection limits, 30-second response times, and the ability to analyze samples with an average error of under 10%. The inclusion of a fluorescent compound as a reference material to compensate for fluctuations from pulsed excitation sources has further increased the reliability and accuracy of each sensor. Finally, after developing sensors capable of monitoring zinc and copper individually, these sensors are combined to form a single optical fiber sensor array capable of simultaneously monitoring concentration changes in zinc and copper in aqueous environments.

## 1. Introduction

The use of optical fibers as a means for sensing a target analyte has wide ranging applications. Optical fiber sensors have been employed to monitor physical characteristics such as strain or temperature changes [1,2]. Of greater pertinence to this research is the work that has been done in the past to detect the presence and determine the concentrations of various chemicals or biomolecules [3,4]. Analytes such as pH [5], dissolved oxygen [6], and carbon dioxide [7] have been well studied and a variety of sensors have been developed which are able to quantitatively analyze solutions for these parameters. However, a category of analytes which has not received as much attention for detection with optical fiber-based sensors, are transition metal ions. While some single-metal sensors were developed in the past [8–10], multi-analyte optical fiber sensors have not been extensively investigated. Our crossed-fiber design allows for fabrication of sensor arrays capable of analyzing aqueous environments for metal ions (here copper and zinc) and for spatially resolved readout of these arrays with optical time of flight spectroscopy (OTOFS) [11].

### 1.1. The Need for Zinc and Copper Monitoring

Zinc and copper are commonly occurring metals and are used in a variety of processes. Because of its ability to resist corrosion and the capacity to effectively bind with many other materials, zinc constitutes an ideal anti-corrosion material and is commonly applied via electroplating methods [12]. Zinc is also commonly used in metal alloys, with brass, a mixture of copper and zinc, being the most common. Copper is the third most commonly used metal in industrial processes [13]. Most electrical wiring within commercial and residential structures is copper-based. Lastly, most household plumbing applications that employ metal pipes are also made of copper.

With the many different applications for both of these metals, introduction of either metal into environmental settings can occur via a variety of pathways. Many different point sources have been identified as polluting species. These can include mining [14], extraction processes used to refine the metals [15], and industrial wastewater generated from various processes [16]. However, non-point sources can also contribute to the introduction of these species into the environment. Storm runoff or leaching from landfills may contaminate the local waterways [17]. Because of all of these potential sources of pollution, the need for accurate and reliable means of analysis is required to assist in preventing these species from entering regulated systems or containing them once introduced.

While both of these metals are required for physiological processes, elevated exposure to either metal has been proven to be detrimental. While short-term exposure to elevated levels of either copper or zinc has not been shown to result in any detrimental health effects [18,19], ingesting elevated levels (above 11 mg/kg/day) of zinc can cause serious adverse effects on the gastrointestinal system, inducing strong nausea, vomiting or intestinal bleeding. The daily amount of copper required (0.013 mg/kg/day) is usually obtained through a normal diet and excess copper is most often introduced through the drinking water supply due to copper leaching from household plumbing. Exposure to elevated levels of copper is also indicated by strong gastrointestinal distress, usually vomiting or nausea. Studies have found that a concentration of 4 mg/L of copper will cause nausea, while 6 mg/L will induce vomiting.

While there are other methods currently available for copper and zinc analysis, these methods suffer from one of two shortcomings. For traditional laboratory-based instrumentation such as flame atomic absorption spectroscopy (FAAS) or inductively coupled plasma-mass spectrometry (ICP-MS) [20,21] accuracy and precision are achieved at the cost of rapid, onsite analysis for industrial facilities. On the other hand, more portable methods such as colorimetric test kits [22], lack the precision and accuracy required for quantitative analysis. The sensors described here are designed to bridge the gap, providing accuracy and reliability near the levels achieved by standard bench top methods while providing rapid feedback capability.

### 1.2. OTOFS and the Crossed Fiber Array

Optical time of flight spectroscopy using optical fibers was first described by Browne, *et al.* in 1996 [23] for quasi-distributed fluorescent sensor regions along a single optical fiber. For readout of theses sensors an excitation light pulse is coupled into the front end of the fiber. The pulse travels in the fiber to sensor regions, where the fluorosensors are excited. The resulting fluorescence pulses emitted from these fluorosensors return through the same fiber to its front end. Because the fluorescence pulses emitted by fluorosensors located farther from the front end of the fiber arrive with a characteristic time delay at the detector, the location of the emitting sensor region on the fiber can be calculated from this time delay. In order to temporally resolve the fluorescence pulses from adjacent sensor regions, these must have a minimum spacing on the fiber, which for typical fluorophores with nanosecond fluorescent lifetimes is of the order of 5–10 m.

In most quasi-distributed optical fiber sensors, the sensor regions are created in the fiber cladding or in a suitable substance replacing this cladding. To avoid refractive losses at sensor regions (*i.e.*, to maintain conditions of total internal reflection), the refractive index of a replacement cladding needs to be equal to or less than the refractive index of the original cladding (assuming that it is retained outside of the sensor regions). In this case, the excitation of the fluorosensors in the cladding is through the evanescent fields of the fiber-core modes; likewise, the fluorescence pulses of the sensors are captured via the evanescent fields into a core mode, which will propagate under guided conditions to the fiber ends.

**Figure 1.**
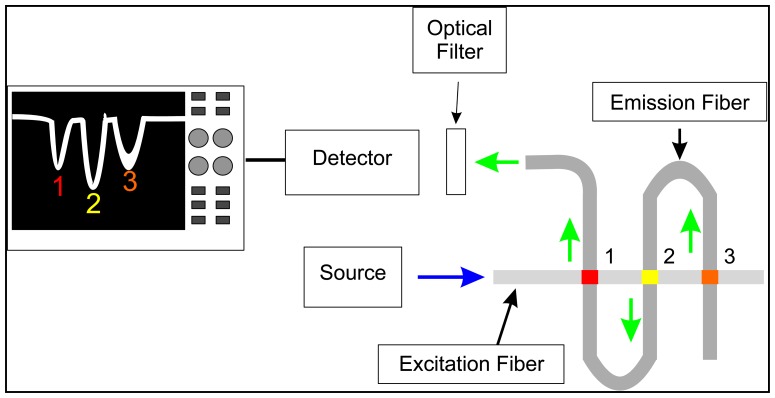
A schematic depicting the experimental design of a crossed-fiber sensor array containing three sensor junctions (labeled 1, 2, and 3). The blue arrow indicates the excitation pulse and the green arrows indicates a fluorescence signal.

While OTOFS is possible using a single fiber, the lengths of fiber required for multi-analyte detection and quantitation may be prohibitive for sensing tasks in confined spaces. In previous research, we demonstrated an optical fiber design utilizing two optical fibers arranged orthogonally to each other, referred to as a crossed-fiber sensor array [24]. An example of this design is shown in Figure 1. Where the two fibers intersect, the cladding and buffer regions for both fibers are removed, exposing the glass cores. Sensor regions are created at these fiber junctions by depositing a polymer doped with fluorescent sensor molecules in place of the original cladding and buffer, and joining the two fibers together. Many such junctions can be created for different sensing tasks (e.g., the presence of specific compounds), for redundancy and for general environmental and experimental conditions (e.g., temperature, pH, excitation pulse intensity, *etc.*) that may affect the response of the primary sensors. As in single-fiber OTOFS, a light pulse travels through one of the fibers (referred to as the excitation fiber) to the sensor junctions, where the fluorosensors are excited via evanescent fields. The fluorescence pulses, however, are captured via evanescent fields of the second fiber (*i.e.*, the emission fiber) and travel through this fiber to a detector (in this case a PMT) located at the end of this fiber. Along the emission fiber, the sensor regions are spaced by a sufficient length of optical fiber for the pulses emanating from all sensor regions to are fully resolved at the detector; along the excitation fiber, these may spaced as close as a fiber diameter, although for practical reasons such as sensor fabrication requirements, the actual spacing is usually a few cm. One advantage of the crossed-fiber scheme is that instead of requiring the extra lengths of optical fiber to be located between each of the sensors, the excess fiber can now be stored off to the side as part of the emission fiber, allowing for a large sensor density at a measurement site. Moreover, there is a significant background reduction, because in the ideal case only fluorescence and not excitation light is captured by the emission fiber; however, while in practice some cross-coupling of excitation light does occur, the achieved background reduction still constitutes a significant improvement.

All of the research described herein utilized the crossed-fiber sensor array design. The goal was to create separate sensor regions for zinc and copper ions in aqueous solutions. The chosen sensor molecules respond to the presence of the target ions by a change in the fluorescence intensity: the zinc sensor molecule increases the intensity, whereas the copper sensor decreases the intensity with increasing concentration of the respective analyte. A precise and accurate measurement thus requires that other effects that may lead to a change in the emissions intensities are taken into account. In the array platform, this is accomplished by pairing each metal-ion sensor with another sensor junction that measures the excitation pulse energy (while being unaffected by other environmental influences). This allows for referencing the measured metal-ion sensor signal to the corresponding pulse energy, thus removing pulse-energy fluctuations originating at the laser itself, but also during the propagation of the pulse from the source to the sensor regions. Thus, the sensor array described here consists of four sensor junctions.

## 2. Experimental Setup and Materials

### 2.1. Experimental Setup

A Photon Technology International (PTI, Birmingham, NJ, USA) PL-3300 nitrogen laser was used to pump a PL-201 tunable dye laser (also PTI). To monitor both pulse intensity and to mark the timing of pulse generation from the laser, a Thorlabs (Newton, NJ, USA) DET10A fast photodiode, with a rise time of 1 ns, was employed. Emitted pulses were coupled into a FT-1000-UMT, high OH optical fiber (NA = 0.39) from Thorlabs and transmitted to and coupled into the fiber-sensor array used for measurements. Excitation pulses were generated at a wavelength of 495 nm using a 1.0 × 10^−2^ M solution of the dye Coumarin-481 in *p*-dioxane and at 365 nm using a 6.0 × 10^−3^ M solution of 2,5-diphenyloxazoleintoluene. Both dyes were purchased from Exciton (Dayton, OH, USA) and the solvents were purchased from Sigma-Aldrich (Milwaukee, WI, USA).

The optical fibers utilized in the sensor arrays were polymer-clad with a core diameter of 200 μm and a numerical aperture of NA = 0.39 (ThorLabs FT-200-UMT). The emission fiber was terminated with a removable SMA connector, which was used to connect this fiber to a 0.5-inch-diameter tube which in turn contained either a FL514.5-10 narrow bandpass filter centered at 517 nm with a bandwidth of 7 nm for zinc measurements or a B52-544 yellow dichroic 500-nm longpass filter for copper measurements (Edmunds Optics, Barrington, NJ, USA). The sensor-fluorescence signal passed through the filter before reaching the detector.

Both excitation and emission fibers had a 0.75-cm section of cladding and buffer material removed in order to expose the glass cores. The removal process was accomplished by placing the optical fiber on a metal sheet drilled with small (1-cm diameter) holes. The optical-fiber section exposed by the hole was then placed over a Bunsen burner and the buffer/cladding materials were burned off. Care had to be taken during the removal process as the fibers were significantly more brittle if heated until a red glow was observed. These fibers were laid orthogonal to each other and then secured between two polypropylene blocks, which at their centers had a 1.25-cm-diameter hole to allow for analyte access to the fluorescent sensor; the sensor junctions were located at the center of this hole. These blocks provided additional support, which was required because the fibers are quite fragile once the glass cores are exposed. After securing the fibers, a liquid polymer solution, serving as cladding in the sensor junctions and containing the desired sensor dye, was added dropwise to the intersection point of the fibers and cured with a handheld UV lamp operating at wavelength of 365 nm. This polymer, once cured, kept the fibers in a junction at a fixed distance, which is crucial because of the exponential decay of the evanescent fields with distance normal to the fiber cores: any shift in relative position between the two fibers could cause significant changes of signal intensities, thereby increasing the uncertainties in the concentration measurements of the analytes.

The sensor signals were detected with a Peltier-cooled Burle C31034A photomultiplier tube with an applied voltage of 1.8 kV. In order to provide a more accurate measurement, 100–300 individual fluorescent signal pules were collected and averaged using a LeCroy LC 564 DL 1-GHz oscilloscope (Chestnut Ridge, NY, USA). The data was transferred to a PC using ScopeExplorer, ver. 2.25 (LeCroy). Data processing was accomplished using a combination of Origin 7.0 and Microsoft Excel 2010. In order to increase the reliability of data analyzed, it was determined that integrating the area under the peak of the fluorescence signal was more precise than only utilizing the maximum value associated with the peak of the pulse.

While integrating the area under each peak did increase the precision of the sensor, there was still a significant source of variability present as the pulsed nitrogen laser used as the excitation source exhibits pulse-to-pulse energy fluctuations. This variance in pulse energy will induce changes in the fluorescence intensity generated by a sensor, which are not related to changes in analyte concentration and for which a correction must be applied. Two different methods were developed in order to correct for this issue, one utilizing a fast photodiode located at the laser source and another utilizing a second crossed-fiber sensor acting a reference. The details of these corrective measures will be discussed for both the copper and zinc sensors in the experimental section. Regardless of reference type, the data collected was also integrated in a manner identical to that of the analyte-specific sensors.

### 2.2. Chemicals Employed

FluoZin-1 tripotassium salt, used as zinc-sensing dye, was purchased from Invitrogen (Carlsbad, CA, USA) and was dissolved in ultrapure water. Dragon Green fluorescent microspheres and polymethylmethacrylate (PMMA) microspheres were purchased from Bangs Laboratory (Fisher, IN, USA). Quinine sulfate monohydrate, polyethylene glycol diacrylate (PEG-DA), 2,2-dimethoxy-2-phenylacetophenone (DMPA), trimethylolpropane triacrylate (TPT), dansyl chloride, diethylenetriamine, 3-glycidoxypropyltrimethoxysilane (GPTS), acetonitrile, acetone and toluene were purchased from Sigma Aldrich. Ultrapure water with a resistivity of 18 MOhm·cm was prepared using a Thermo-Scientific Nanopure Ultrapure Water Purification System (Waltham, MA, USA).

### 2.3. General Instrumentation

Absorption spectra were collected using both a Perkin-Elmer (Waltham, MA, USA) Lambda 650 UV/Vis Spectrophotometer and an Agilent 8453 UV-Visible Spectrophotometer system (Santa Clara, CA, USA). Fluorescence spectra for the determination of spectral properties of the sensors were recorded using a Fluorolog-3 FL-22 flourimeter made by Horiba Scientific (Ann Arbor, MI, USA).

## 3. Experimental Results

### 3.1. Zinc Sensor

**Figure 2.**
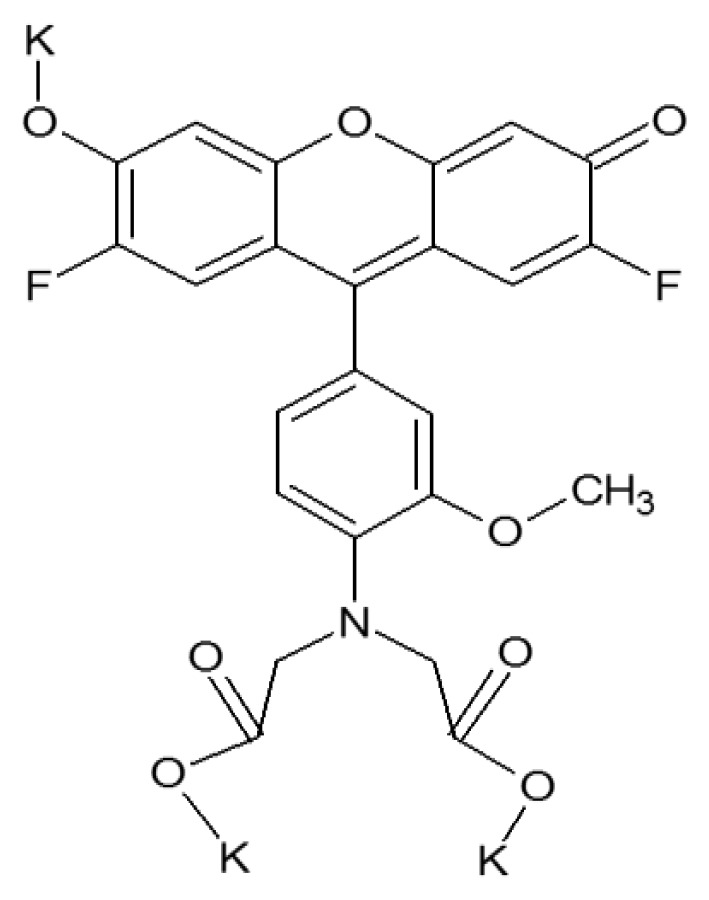
The chemical structure of FluoZin-1.

For zinc recognition the commercially available compound, FluoZin-1 (FZ-1. Figure 2) was employed [25]. This fluorophore was chosen because it was stated to measure zinc between 0.05 and 3.30 mg/L. The discharge limit set by the EPA, which states that a manufacturing facility may discharge up to an average of 1.48 mg/L of zinc per day over a 30-day period, falls within this range. Moreover, FZ-1 has excitation and emission wavelengths (λ_ex_/λ_em_ = 495 nm/515 nm) for which the optical fibers used here have a high transmittivity, which is important for potential remote deployment. Upon binding with zinc, FZ-1 exhibits fluorescence enhancement because of the inhibition of the photo-induced electron transfer process.

**Figure 3.**
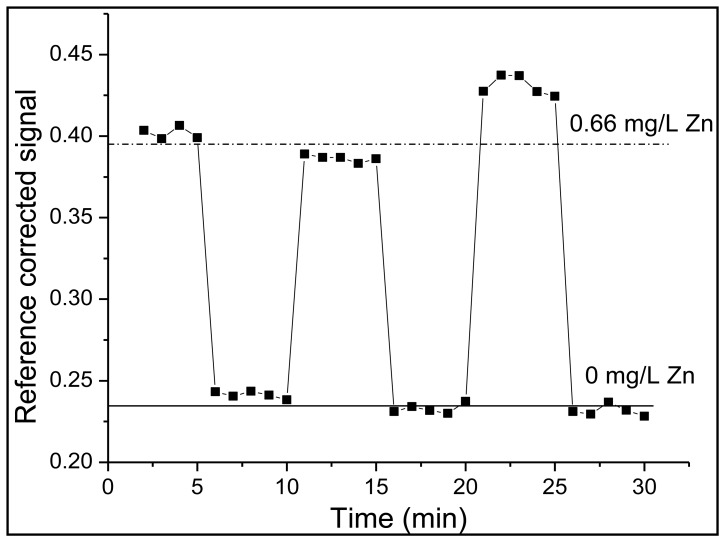
Rapid response time to changes in zinc concentration by FZ-1 in micro-templated polymer matrix.

To integrate FZ-1 into the polymer encapsulating the sensor junction, it was dissolved in ultrapure water and mixed into a polyethylene-glycol-diacrylate-based polymer solution to yield a final FZ-1 concentration of 4.17 × 10^−5^ M. The polymer solution consisted of three components. While PEG-DA, a cross-linking polymer, was the main component, DMPA was added in order to induce photo-polymerization via a free-radical reaction [26]. TPT served as an additional cross-linking agent in order to increase the structural rigidity of the polymer and limit the rate at which FZ-1 would leach from the cured polymer matrix. This procedure produced a rigid polymer matrix, which surrounded the sensor's dyes. Thus, the response time of the sensor is determined by the density of the polymer matrix, which in turn affects the diffusion of zinc ions through the polymer matrix to sensor molecules sites. The response time was found to be of the order of 90 min, which is prohibitive for real-time wastewater monitoring. This can be improved by creating a more porous polymer matrix; however, this will lead to swelling of the polymer, which will affect the signal intensities because of concomitant changes in the separation of the two fibers. An alternative way of increasing the flow rate of the analyte through the polymer to the sensor molecules is to engineer microscale channels into a rigid (*i.e.*, high density) scaffold. This can be accomplished with a technique called micro-templating [27]. A sacrificial template, in this case solid PMMA microspheres with a diameter of 10 μm, are mixed into the liquid polymer solution with an identical composition as listed above. After the polymer mixture is added to the optical fiber sensor junction, the polymer is cured and solidifies. This fiber-sensor junction is then placed in acetone, which dissolves the microspheres, leaving a continuous network of channels within the polymer [24,26]. This allows for the rapid transit of the analyte to the sensor fluorophore. Subsequent trials demonstrated a reduction in response time from 90 min to 30 s, when zinc concentrations either increased or decreased, as shown in Figure 3.

**Figure 4.**
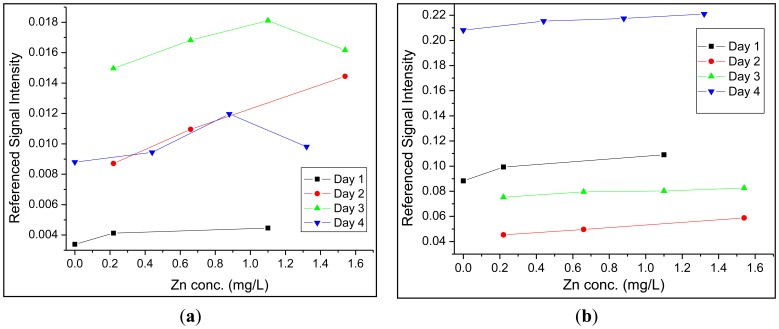
Calibration curves collected over multiple days (**a**) utilizing a fast photodiode as the excitation intensity reference, (**b**) utilizing a second cross-fiber sensor as the excitation intensity reference.

Up to this point, a single sensor region was employed to analyze solutions for zinc. However, the issue of source pulse energy fluctuations still needed to be addressed. First, the fast photodiode located at the excitation source, which measured back-scattered radiation from the front face of the optical fiber when a pulse was injected, was employed for this purpose. For both detectors, 100–300 signals were averaged to provide one trace on the oscilloscope. The area under each peak in a trace was integrated and a ratio between the integrated fluorescence and the integrated back-reflected signal was determined. This provided a means of correcting for fluctuations in laser pulse intensity discussed in Section 2.1. The second approach was to employ a fluorescent reference sensor in close proximity to the site of the primary measurement (*i.e.*, at the site of the zinc sensor). This method was expected to yield further improved figures of merit, because this method should also account for additional excitation-pulse-energy fluctuations that may arise as these pulses travel from the laser source to the sensor sites. The two principal requirements for fluorophores that are to be used as reference sensors are (1) that the fluorophore must have an identical or similar spectral profile and (2) that these must be resistant to chemical changes in solution such as a change in pH or a change in the concentration of metals present. Dragon Green, the trade name for commercially available polymer microspheres doped with a fluorescent photostable dye, was determined to meet these characteristics (λ_ex_/λ_em_ = 495 nm/515 nm). These fluorescent microspheres were included within the replacement-cladding polymer in a manner identical to FZ-1. While none of the figures of merit showed significant improvement when compared to the fast photodiode reference, a marked increase in the reliability between day to day measurements was observed for the experimental setup which utilized FZ-1 and Dragon Green in the crossed-fiber array. Over a period of four days, calibration curves were collected using both methods of referencing (Figure 4). Using Dragon Green as a reference sensor in the crossed-fiber array provided linear calibration curves over multiple days, thus maintaining the linear relationship between the concentration of zinc and fluorescence intensity. When examining the calibration curves generated using the fast photodiode, it was noticed that a linear relationship was only obtained 50% of the time. Data analysis utilizing no reference signal (photodiode or optical fiber-based) revealed similar trends to those seen in Figure 4a, but the non-referenced data exhibited larger variances for each data point. This further supports the need for a second optical-fiber based sensor to serve as an intensity reference.

Figures of merit for the zinc sensor were determined following published methods and are summarized in Table 1. The limit of detection (LOD) and limit of quantitation (LOQ) were determined using previously published methods [28]. The LOD was determined to be 0.02 mg/L, whereas for the LOQ a value of 0.05 mg/L was obtained. While the concentration of FZ-1 could be varied in order to adjust the linear dynamic range, the FZ-1 concentration of 4.17 × 10^−5^ M provided a linear dynamic range (0.05–1.50 mg/L), which encompassed the EPA discharge limit. The three-week lifetime for each sensor was due to leaching of FZ-1 from the polymer into solution. In order to generate maximum levels of fluorescence emission, the pH was held at 5.5 using a sodium acetate/acetic acid buffered solution and the sensor was stored in ultrapure water when not in use. Lastly, it was determined that FZ-1 did recognize three other elements (lead, cadmium, nickel) but FZ-1 showed a selectivity for zinc when exposed to both zinc and one of the other species simultaneously.

With this two-sensor array, a series of samples containing of unknown levels of zinc were analyzed. Each sample was divided in half and the first half of the sample was analyzed using the optical fiber sensor array. The second half of each sample was sent to a state-certified facility for analysis by FAAS to provide independent values for comparison. After collecting the data it was found that the five out of eight samples analyzed provided statistically similar values between the two methods of analysis. For these five samples, the % difference between the two values was less than 8%. This data is shown in Table 1. For the samples, which failed the t-test, a higher number of data points collected may resolve this issue. In order to maintain the rapid analysis capability, the number of pulses averaged for each data point must be reduced but earlier experiments have shown that 100 pulses should be sufficient for monitoring [29].

### 3.2. Copper Sensor

**Figure 5.**
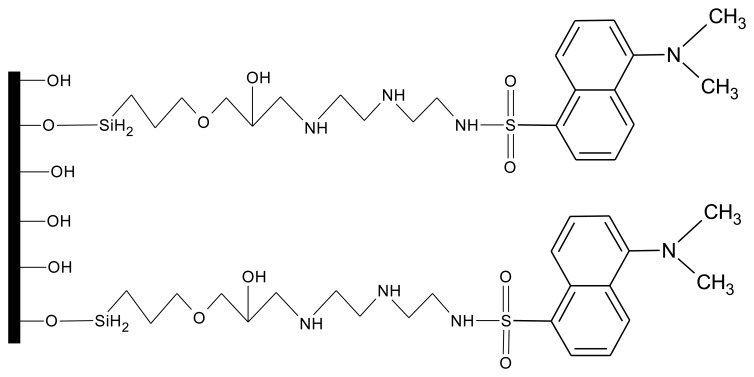
The chemical structure of DDETA, bound to a glass surface.

While the zinc sensor, FZ-1, had to be mixed into the polymer mixture that was added to the crossed-fiber junction, for the copper-specific fluorosensor an alternative approach was chosen. Using a process outlined by Ding *et al.* [30] the copper sensor molecule (referred to as DDETA) was covalently attached to the surface of the core of the optical fiber (Figure 5). Again, great care had to be taken when handling the fibers as they were very brittle after removal of the original cladding material. This covalent scheme eliminated the drawback of dye leaching noted with the zinc sensor and allowed for a more efficient excitation of the fluorophores via the evanescent waves. While FZ-1 underwent fluorescent enhancement upon binding of zinc, fluorescence quenching (λ_ex_/λ_em_= 365 nm/490 nm), due to a twisted internal charge-transfer mechanism, was observed when copper was bound to DDETA. While not required for sensor molecules covalently attached to the fiber-core surface, a micro-templated polymer cladding was still employed in order to keep the two fibers in constant proximity to each other. This was applied after the covalent attachment of the sensor dye.

DDETA recognizes copper by initially binding copper at the dimethylamino site located at the end of the molecule chain that was attached to the optical fiber surface. Upon binding, the other nitrogen atoms help to stabilize and strengthen the binding of copper to DDETA. Due to steric hindrances that occur because of the bulky dansyl group, the loading of DDETA molecules on the fiber-core surface is limited. This will restrict the concentration of copper ions that can be monitored to low (<0.5 mg/L) copper concentrations.

**Figure 6.**
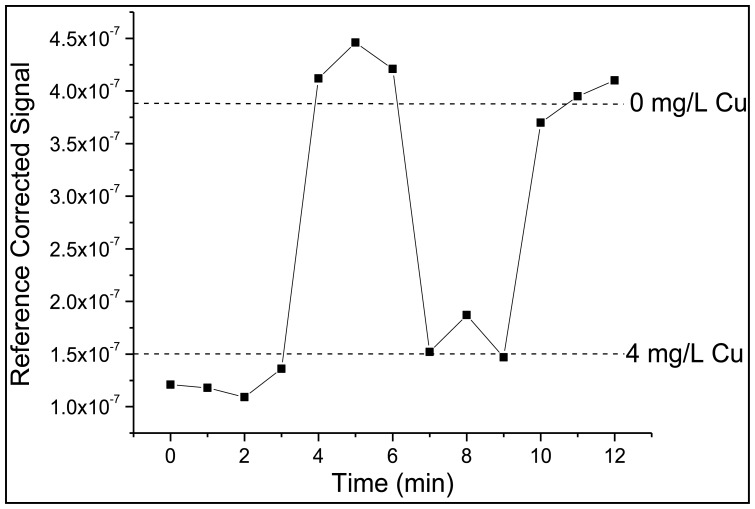
Rapid signal stabilization after changes in copper concentration.

The copper sensor was evaluated and validated in the same manner as zinc, using the fast photodiode as the reference for initial studies. It should be noted that response times are once again on the order one minute or less (Figure 6). However, while the zinc sensor could be immersed in ultrapure water to remove zinc from FZ-1, DDETA required immersion in a 0.05 M EDTA solution with a subsequent ultrapure water rinse in order to remove the bound copper. While the limit of detection (0.18 mg/L) and limit of quantitation (0.68 mg/L) are higher than observed for the zinc sensor the linear dynamic range is ideal (0.68–7.00 mg/L) as it encompasses the EPA limit for wastewater copper discharge, in this case 2.07 mg/L. DDETA also demonstrated an ability to recognize zinc; this issue will be addressed in Section 3.3.

**Figure 7.**
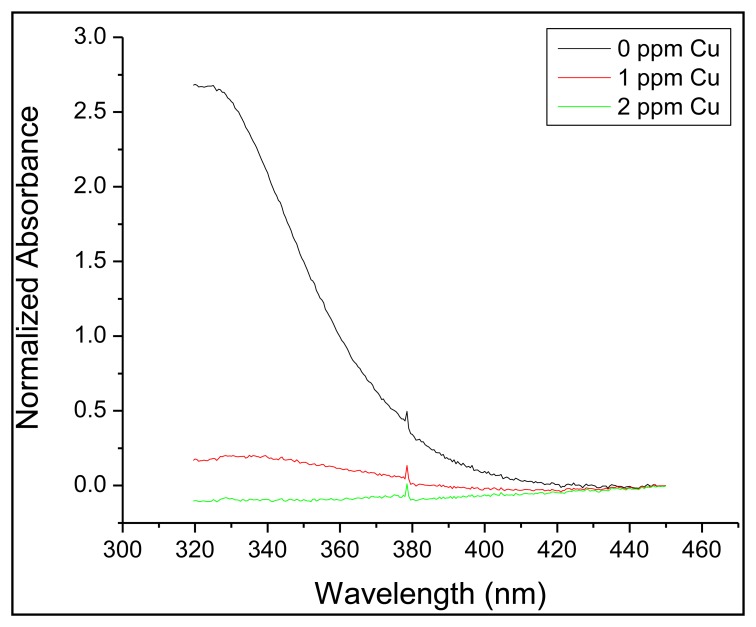
The UV-Vis absorption profile of DDETA attached to a cuvette when exposed to multiple copper-containing solutions.

As with the zinc sensor, a reference fluorophore was required in order to develop a more effective copper-monitoring platform. In this case, the selected reference fluorophore was quinine sulfate monohydrate. However quinine sulfate also experiences fluorescence quenching in the presence of copper. To correct for this, quinine sulfate was encapsulated in polyacrylonitrile using a previously established method [31]. This process generated an impermeable shell around the quinine sulfate, preventing copper or any other species from influencing the fluorescence of quinine sulfate. In order to confirm that the quinine sulfate was encapsulated, solutions of both non-encapsulated and encapsulated quinine sulfate were exposed to 1 mg/L solutions of copper. It was observed that the percent change in fluorescence between a clean solution and a copper-containing solution was greatly reduced when comparing the non-encapsulated (27%) against the encapsulated (2%) quinine sulfate. Because DDETA undergoes fluorescence quenching, and the covalent attachment scheme allowed for stable baseline fluorescence intensities for >4 weeks, an alternative approach to daily calibration was pursued. Dynamic or static quenching are, in many cases, described by the following Equation [32]:

(1) $$\frac{I_{0}}{I}=1+K_{\text{Q}}[Q]$$

where [*Q*] is the quencher concentration, and *I* and *I*_0_ are the measured fluorescence intensities in presence and absence of the quencher, respectively. For dynamic quenching this equation is referred to as the Stern-Volmer equation for collisional quenching, and the quenching constant, *K*_Q_ (now referred to as the Stern-Volmer coefficient, *K*_SV_) is determined by the unquenched lifetime of the excited state of the fluorophore, and also contains, for fluid media, the diffusion-limited rate constant for the formation of the fluorophore-quencher encounter complex. For static quenching, in which the fluorophore forms a stable ground-state complex with the quenching species, the quenching constant *K*_Q_ is now the association constant, *K*_Q_, of that complex. For DDETA it is expected that static quenching is dominant, which is supported by the fact that DDETA required a 0.05 M EDTA solution to regenerate the sensor by removing the copper from the sensor. Examining the UV-Vis absorption spectrum of DDETA in the absence or presence of copper further supports that static quenching is the dominant mechanism. Because static quenching occurs when a ground-state complex forms, the absorption profile is expected to change. Figure 7 shows that the principal absorption peak at 330 nm disappears as the concentration of copper is increased, suggesting that the formation of the DDETA-copper complex is occurring. For systems undergoing dynamic quenching, the absorption profile should not change, as the interaction between analyte and fluorophore occurs while the fluorophore is in the excited state. While static quenching is believed to be the principal quenching mechanism, there is the possibility that a dynamic quenching contribution is also present. Further conclusive confirmation of a static quenching mechanism may be furnished by the examination of the fluorescence lifetime (τ_0_), which should exhibit no change in the presence of quenching species, and by determining the temperature dependence of *K*_Q_, which should be inversely related [33]. However, complete characterization of the fluorescence quenching mechanism was beyond the scope of this work. To determine *K*_Q_, calibration curves were collected, one each day. For each day a value for *K*_Q_ was determined, and then these four values were averaged to give a final constant. Four and five days later, after collecting the baseline fluorescence (*I*_0_), several simulated samples were analyzed without performing a calibration curve. The fluorescence intensity was then inserted into Equation (1) and the copper concentration was determined. As seen in Table 2, 6 out of the 7 samples were statistically similar when comparing the calculated value against the expected value.

### 3.3. Four-Sensor Array

After separately developing the copper and zinc sensors along with respective reference sensors, the four sensors were combined into one sensor array. The order of the sensors with respect to the excitation source was DDETA, quinine sulfate monohydrate, Dragon Green and ending with FZ-1. This order was chosen because excitation pulses required for the copper sensor and its reference (at a wavelength of 365 nm) will suffer more attenuation losses over equal lengths of fiber than the 495-nm wavelength excitation pulses needed for the zinc sensor. Thus, this ordering maximized the excitation intensity at each of the sensors, regardless of excitation wavelength. In order to separate the signals in time, four separate emission fibers with different lengths were used the four crossed-fiber sensor regions. The difference in length will separate of the emission signals in time, causing all emission pulses to arrive sequentially at the (single) detector. To achieve this, the emission fiber of a given sensor was chosen to be 10 meters longer than the fiber for the previous sensor. The lengths of emission fibers were as follows: DDETA—0.5 m, quinine sulfate—15 m, Dragon Green—25 m, FluoZin-1—35 m.

**Figure 8.**
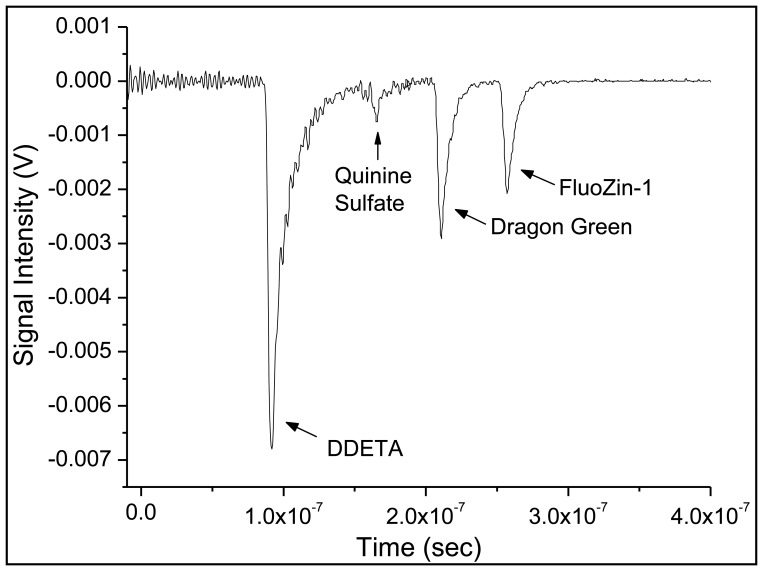
The output trace collected from an oscilloscope depicting the detection of every sensor in the four-sensor array in time. The zero point in time indicates excitation pulse generation.

Collection of the data was a two-step process because two different excitation wavelengths were required for the sensors. The set of copper-sensor signals (reference and analyte-specific) were recorded first. Then the excitation wavelength and emission filter were changed in order to measure the set of zinc-sensors signals analysis. These two traces were combined to generate the Figure 8, using the same time reference—the firing of the laser pulse—for both traces. No rescaling of the traces was done before combing them into a single graph, nor were the baselines shifted. Moreover, by measuring the difference in time (Δ*t*) between two peaks, the change in the length of emission fibers, *d*, can be calculated using the Equation (2):

(2) $$\frac{c}{\eta_{\text{core}}}*\Delta t=d$$

where *c* is the speed of light and *η_core_* is the refractive index (*η_core_* = 1.46) of the fiber-core utilized in the array. This provided a means of confirming from which sensor region the signal emanated. The large gap between the laser pulse at *t* = 0 and the first recorded fluorescent pulse is due to the 30 m of fiber between the source and the first sensor region.

**Figure 9.**
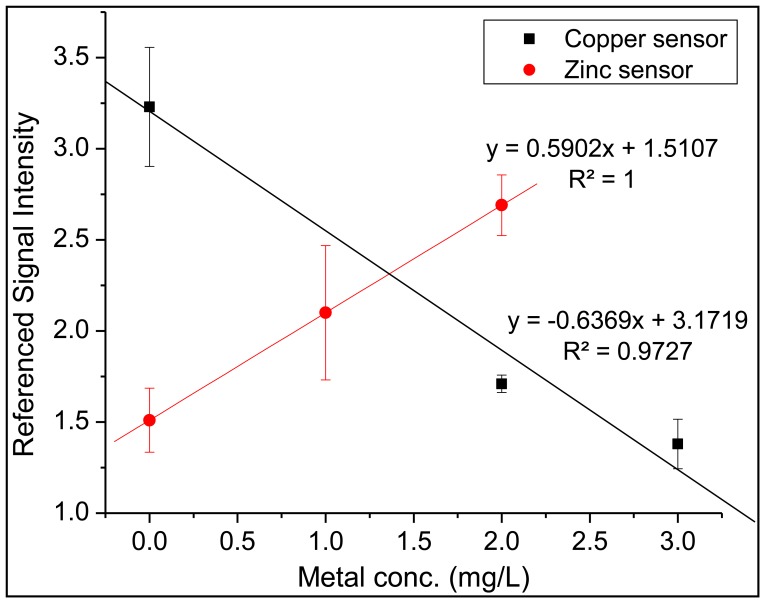
An analysis of solutions containing both copper and zinc in the same solution with the four sensor array.

After confirming the successful recognition for each sensor region, the sensor array was placed in a 9 × 13 inch Pyrex^®^ baking dish and solutions containing both zinc and copper were added. Measurements were collected in the same manner as listed above, with the copper analysis occurring first, followed by changing the excitation wavelength and emission filter and ending with the zinc analysis. The entire array was immersed in a 0.05 M solution of EDTA for 5 min, then immersed in an ultrapure water bath for five minutes after each analysis cycle. As seen in Figure 9 the trend lines for both sensors indicate that the copper sensor and zinc sensor were able to successfully monitor the target analyte without suffering any detrimental effect from the competing metal species in solution. The metal concentration referred to on the x-axis represents the concentration of each individual metal (*i.e.*, a data point collected at 2 mg/L on the x-axis represents an analyzed solution which contained 2 mg/L of zinc and 2 mg/L of copper in the same solution). It is worth noting the difference in the fluorescence response for each metal-specific sensor. For zinc, FluoZin-1 undergoes fluorescent enhancement as the concentration of zinc is increased due to the inhibition of the photo-induced electron transfer (PET) process [34]. Copper, a known fluorescence quencher, will induce fluorescence quenching after complexing with DDETA [35].

## 4. Conclusions

We have demonstrated the successful creation of a multi-analyte optical fiber-based sensor array. Two sensors, capable of monitoring zinc and copper at concentration levels, which encompass government discharge limits at industrial facilities, were developed. These sensors showed rapid response times to changes in analyte concentration as well as the ability to effectively and accurately analyze samples containing unknown amounts of metal. Lastly, these sensors were combined into a four-sensor array for the monitoring of solutions for both copper and zinc.

While this research has demonstrated the capability of both sensors to determine the concentrations of the respective metals in relevant concentration ranges, several issues need to be addressed before deployment of these sensors in industrial or environmental waters. The need for daily calibration of the zinc sensor will limit remote-sensing capability. Both sensors need to be regenerated by immersion in ultrapure water or EDTA for the zinc and copper sensors, respectively. In industrial facilities, where the environments can be controlled, these issues can be compensated by using designs which utilize flow injection systems or microfluidic devices to provide the appropriate platform in order to allow for sample or sensor treatment.

Moreover, the experiments described here were carried out in a buffered solution to maintain pH = 5.5, where the fluorescence intensity of the zinc sensor has its maximum. For a given zinc concentration, the fluorescence intensity decreases to one half of its maximum value at pH ∼ 4 and pH ∼ 7, with these values establishing a pH over which the sensor can be employed. In a real-world environment, pH fluctuations may occur and the zinc concentration measurement has to be corrected for these. For this purpose a pH sensor can be added to the sensor array; we already implemented such a sensor for our sensor architecture [24].

Also, the current experimental setup is cost and size prohibitive to facilitate deployment, either remotely or in an industrial setting. Use of an alternative, smaller excitation source and the miniaturization of detection components will allow for a more cost effective and space efficient design.

## Figures and Tables

**Table 1.** The average concentration of zinc present in simulated samples as measured by both optical fiber sensor and an independent lab. The data in the table column labeled “statistically similar?” are the result of the Student's t-test to compare the values obtained by the optical fiber sensor against the independent lab.

**Expected Values (mg/L)**	**Optical Fiber Sensor (mg/L)**	**Independent Lab (mg/L)**	**Statistically Similar? (n = 4 @ 95%)**
1.00	0.910 (±0.170)	1.050 (±0.000)	Yes
0.88	0.833 (±0.132)	0.872 (±0.003)	Yes
0.86	0.832 (±0.021)	0.828 (±0.019)	Yes
0.75	0.788 (±0.068)	0.800 (±0.003)	Yes
0.67	0.749 (±0.082)	0.642 (±0.000)	No
0.50	0.442 (±0.037)	0.534 (±0.006)	No
0.47	0.512 (±0.115)	0.491 (±0.004)	Yes
0.25	0.243 (±0.038)	0.277 (±0.002)	No

**Table 2.** A summary of the copper concentrations determined using Equation (1) with a comparison against the expected value. The Student's t-test was used to determine statistical similarity.

**Expected Value (mg/L)**	**Calculated Value (mg/L)**	**% Difference?**	**Statistically Similar (n = 4 @ 95%)**
1.00	1.03 ± 0.08	2.50	Yes
1.68	1.96 ± 0.08	17.24	No
2.00	1.98 ± 0.07	0.10	Yes
2.59	2.64 ± 0.18	1.84	Yes
3.00	3.08 ± 0.07	2.67	Yes
3.56	3.52 ± 0.07	0.88	Yes
4.47	4.14 ± 0.06	7.33	Yes
